# Prescribing at 95 years of age: cross-sectional findings from the Newcastle 85+ study

**DOI:** 10.1007/s11096-022-01454-z

**Published:** 2022-07-30

**Authors:** Laurie E. Davies, Andrew Kingston, Adam Todd, Barbara Hanratty

**Affiliations:** 1 Population Health Sciences Institute, Newcastle University, Newcastle upon Tyne, UK; 2 School of Pharmacy, Newcastle University, Newcastle upon Tyne, UK

**Keywords:** Aged 80 and over, Drug prescription, Primary care

## Abstract

**Background:**

Previous research has examined prescribing amongst 85-year-olds in English primary care, but less is known about prescribing amongst 95-year-olds in spite of population ageing.

**Aim:**

We describe the most commonly prescribed medicines in a cohort of 95-year-olds, using 10-year follow-up data from the Newcastle 85+ Study (n = 90).

**Method:**

A total of 1040 participants were recruited to the Newcastle 85+ Study through general practices at 85-years of age, and 90 surviving participants were re-contacted and assessed at 95-years of age. Prescribed medications from general practice medical records were examined through cross-tabulations and classified as preventative or for symptom control based on their customary usage.

**Results:**

Preventative medications with unclear evidence of benefit such as statins (36.7%), aspirin (21.1%) and bisphosphonates (18.9%) were frequently prescribed.

**Conclusion:**

Future research in a larger clinical dataset could investigate this preliminary trend, which suggests that benefit/risk information for preventive medication, and evidence for deprescribing, is needed in the very old.

**Supplementary Information:**

The online version contains supplementary material available at 10.1007/s11096-022-01454-z.

## Impact statements

- Among people aged 95 years old, our findings suggest that regular medication reviews may be important to ensure appropriate and safe prescribing.
- As populations continue to age, clear practical guidelines are needed on prescribing preventative medications in the very old.

## Introduction

The very old (aged ≥ 85) are the fastest growing age group of many developed countries [1]. Previous research has examined prescribing amongst 85-year-olds in English primary care [2], but less is known about prescribing amongst 95-year-olds [3–5] in spite of population ageing. Often frail, living with multimorbidity, functionally and/or cognitively impaired [6–9], nonagenarians are vulnerable to adverse medication outcomes. With advanced age and such conditions, the remaining life expectancy of this patient group is also limited [10, 11]. The Newcastle 85+ Study is now in the 10th year of follow-up, and according to prescribing models, one would expect prescriptions for 95-year-olds to appreciate the diminishing benefits or rising risks of medications in late life [10, 11].

### Aim

To inform future investigation of clinical data sets to better understand how we can optimise medicines intervention in the very old, we aimed to describe the most commonly prescribed medicines amongst a cohort of 95-year-olds, using data from the Newcastle 85+ Study.

### Ethics approval

The Newcastle and North Tyneside Local Research Committee One approved the Newcastle 85+ Study (Ref: 06/Q0905/2). Written informed consent was obtained from participants, and where people lacked capacity to consent—for example, because of dementia—an opinion was sought from a relative or carer (a “consultee”) [12].

## Method

### Design and setting

The Newcastle 85+ study is a longitudinal population-based cohort study of people born in 1921, aged 85 in 2006 (when the study began) and permanently registered with one of 53 participating general practices in Newcastle upon Tyne or North Tyneside Primary Care Trusts in the United Kingdom [13]. Of the 1040 85-year-olds recruited to the study (2006), 90 surviving participants were re-contacted and assessed at 95 years of age (2016) through multidimensional health assessment in their usual place of residence, inclusive of care homes, and review of general practice medical records. Details of the study have been reported elsewhere [12–14]. The interview schedule and general practice record review proforma can be found on the Newcastle 85+ Study website: https://research.ncl.ac.uk/85plus/, whilst an overview of study recruitment and retention is presented in Online Resource 1.

### Medication data

Data on prescribed medications were obtained from general practice medical records. Over-the-counter medications and prescribed items such as vaccines, wound management products and catheter/stoma products were excluded from this analysis (Online Resource 2) [2]. Medications were coded according to the British National Formulary (70th edition).

### Analysis

Prescribed medications were examined through cross-tabulations and classified as preventative or symptomatic based on customary usage. Preventative medicines describe those customarily used to avert the onset of disease or halt or slow the progression of disease, such as statins, antiplatelets and bisphosphonates. Medication combinations were described through intersecting set plots, and the health and sociodemographic characteristics of participants were examined through cross-tabulations. Frailty was measured using the Fried phenotype [15], and cognitive impairment with the Standardised Mini-Mental State Examination [16].

## Results

At 95-years of age, the Newcastle 85+ Study comprised 90 participants (27 men and 63 women). Of whom, 57.8% (n = 52/90) lived in standard (non-supported) housing, 77.0% (n = 67/87) had four or more diseases, 64.2% (n = 52/81) were pre-frail (whilst 24.7% (n = 20/81) were frail), and 49.4% (n = 43/87) were cognitively intact (whilst 21.8% (n = 19/87) had severe cognitive impairment). The majority were dependent (requiring care less than daily (42.7%, n = 35/82), regularly each day (25.6%, n = 21/82) or 24-hourly (20.7%, n = 17/82) (Table 1).

**Table 1:** Health and sociodemographic characteristics of Newcastle 85+ study participants surviving to 95 years of age

Variable	% (n)
*Sex*	
Male	30.0 (27)
Female	70.0 (63)
*Housing*	
Standard (non-supported)	57.8 (52)
Sheltered	7.8 (7)
Care home	34.4 (31)
*Education (years)*	
0–9 years	65.6 (59)
10–11 years	21.1 (19)
≥ 12 years	13.3 (12)
*Deprivation (IMD)*	
< 25th centile	33.3 (30)
25th-75th centile	45.6 (41)
> 75th centile	21.1 (19)
*Number of prescribed medications*	
0	4.4 (4)
1	1.1 (1)
2–4	15.6 (14)
5–9	51.1 (46)
≥ 10	27.8 (25)
*Dependency (Isaacs’ and Neville’s interval measure)*	
Independent (free from care)	11.0 (9)
Low (needs help less than daily)	42.7 (35)
Medium (needs help at regular times daily)	25.6 (21)
High (needs 24-h care)	20.7 (17)
*Frailty (Fried phenotype)*	
Robust	11.1 (9)
Pre-frail	64.2 (52)
Frail	24.7 (20)
*Cognitive impairment (SMMSE)*	
Normal (26–30)	49.4 (43)
Mild (22–25)	19.5 (17)
Moderate (18–21)	9.2 (8)
Severe (0–17)	21.8 (19)
*Disease groups*	
0	0 (0.0)
1	4.6 (4)
2–3	18.4 (16)
≥ 4	77.0 (67)

Where numbers (n) do not sum to 90 data are missing

IMD = Index of Multiple Deprivation; SMMSE = Standardised Mini-Mental State Examination

**Fig. 1:**
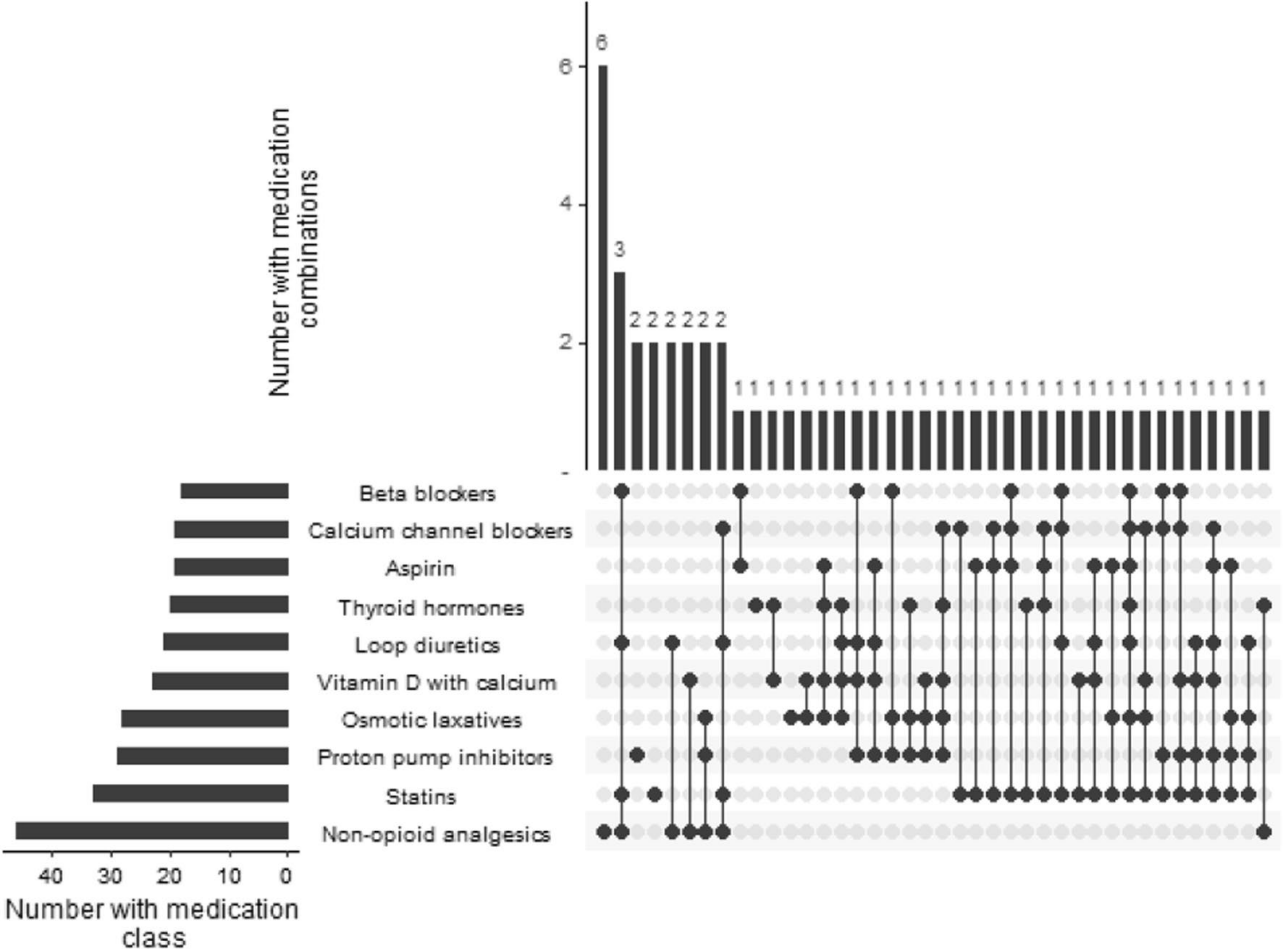
Most common medication combinations amongst Newcastle 85+ study participants surviving to 95 years of age. The upset plot is a graphical representation of the various medication combinations, consisting of three panels: (i) The left-hand panel represents the number of people prescribed that medication; (ii) the bottom-right panel highlights the medication combinations by connected nodes, and (iii) the top-right panel shows the number of people with those medication combinations. For example, over 40 people were prescribed non-opioid analgesics, and of these, 3 people were prescribed non-opioid analgesics with statins, loop diuretics and beta-blockers

Participants were prescribed a mean of 7.4 medications (sd = 3.8). Statins were the most commonly prescribed customarily preventative medication (36.7%, n = 33/90) (Table 2). A variety of medication combinations were prescribed (Fig. 1).

**Table 2:** Most commonly prescribed medicines amongst Newcastle 85+ study participants at 95 years of age

Medication	% (n)
Non-opioid analgesics	51.1 (46)^a^
Statins	36.7 (33)^b^
Proton pump inhibitors	32.2 (29)^a^
Osmotic laxatives	31.1 (28)^c^
Vitamin D with calcium	25.6 (23)^b^
Loop diuretics	23.3 (21)^a^
Thyroid hormones	22.2 (20)^a^
Calcium-channel blockers	21.1 (19)^b^
Aspirin	21.1 (19)^b^
Beta-blockers	20.0 (18)^b^
Bisphosphonates	18.9 (17)^b^
Vitamin D without calcium	18.9 (17)^b^
Oral anti-coagulants	17.8 (16)^b^
Stimulant laxatives	16.7 (15)^c^
Tricyclic and related antidepressants	15.6 (14)^a^
Opioid analgesics	15.6 (14)^a^
Oral iron	15.6 (14)^c^
Angiotensin-converting enzyme (ACE) inhibitors	14.4 (13)^b^
Selective Serotonin Reuptake Inhibitors (SSRIs)	13.3 (12)^a^
Prostaglandin analogues without timolol	13.3 (12)^a^

^a^‘Symptom control’

^b^‘Preventative’ or

^c^‘Both’, based on customary usage

## Discussion

### Statement of key findings

In a cohort of 95-year-olds (n = 90), preventative medications such as statins (36.7%), aspirin (21.1%) and bisphosphonates (18.9%) were commonly prescribed.

### Strengths and weaknesses

Our study extends the limited research on prescribing amongst 95-year-olds [3–5], inclusive of those with cognitive impairment and in care homes, using medication data from general practice medical records as opposed to the less reliable method of self-report [12]. Our data source (the Newcastle 85+ Study) also provides rich contextual information unavailable in other primary care or prescribing datasets, for example, on dependency and care received at home. However our work has limitations. We could not assess the appropriateness of the medications we outline (e.g. statins) according to individual patient circumstances, but there is a lack of clinical trial data in the very old, and in late life a proposal to consider potential medication benefits in relation to estimated remaining life expectancy and (more palliative) care goals [11]. As data were collected in 2016, some of the medication prescribed (for example aspirin over clopidogrel) may relate to out-of-date prescribing practices rather than deprescribing barriers [17]. Whilst 95-year-olds from this study in North-East England are likely to be representative in terms of sex, residential status, ethnicity and multimorbidity, we cannot confirm whether our results apply to other regions of the United Kingdom, for example affluent areas in the south or areas with greater ethnic diversity. The final limitation is the small sample size of the surviving Newcastle 85+ Study participants (n = 90), though the preliminary trends we identify could be investigated in a larger clinical dataset as was our intention.

### Interpretation

Statins are poorly evidence-based in the very old particularly for primary prevention [18]. In this circumstance they take several years to benefit so may be unsuitable at 95 years of age, particularly in those with frailty, cognitive impairment or complex multimorbidity whose remaining life expectancy may be less than one year [11]. Long-term daily aspirin carriers a heightened risk of bleeding in older people [19]. Bisphosphonates too may be unsuitable at age 95, as deprescribing discussions are recommended after three-years of continuous treatment, for example [20].

The prescription of customarily preventative medicines of unclear benefit in late life is widely reported [21] and potential reasons for this include the multitude of deprescribing barriers, such as the lack of evidence for this task, the association with patient-perceived withdrawal of care, fragmented care and fear of negative consequences [22, 23]. There is a danger that withholding preventative medicines from older people, because of limited life-expectancy, can become a self-fulfilling prophecy. For instance withholding anticoagulation may lead to deterioration, given that the risk of stroke from atrial fibrillation significantly increases with age [24].

Several other medication classes most frequently prescribed in this study (Table 2) are considered ‘high risk’ as is often the case in primary care [25]. Loop diuretics (23.3%) have been linked to unplanned hospital admissions in younger populations, for example [26]. But so too has their under-prescription. Their absence can cause breathlessness in heart failure, and under-prescribing is an important problem [27]. Yet it receives less attention, as the harms of inaction are often not as visible in healthcare. It seems we feel more responsible for acts of commission than acts of omission [28], and place more weight on things that come to mind more easily [29] – i.e. an adverse drug reaction-induced hospital admission. Customarily ‘preventative medicines’ such as beta-blockers (for angina or rate control in atrial fibrillation), ACE inhibitors (for heart failure) and calcium channel blockers (for angina) could also be prescribed for symptom control. In which case, the deprescribing of these medications may not be warranted. The variety of medication combinations prescribed to the 95-year-old participants (Fig. 1) further illustrates that ‘one size does not fit all’ when it comes to polypharmacy [2, 30].

Preventative medication prescription in late life is the focus of much research, but symptom control medications such as opioids are notorious as a source of adverse events, often implicated in falls for example. Proton pump inhibitors are also believed to be overprescribed [31], and might in the very least contribute to the burden of medication management often affecting older people [32].

All this considered, our findings suggest that individually-tailored prescribing is needed in the very old, along with evidence of risk-benefit from clinical trials that include older people with multiple conditions and polypharmacy. As to whether deprescribing is the right thing to do in older people, a limited evidence base suggests that it is not harmful in the main and might be beneficial [33, 34]. In the absence of a recent acute coronary syndrome or cerebrovascular event, the discontinuation of a statin toward the end of life may be reasonable [10], and Kutner and colleagues recently concluded that statins can be deprescribed safely and potentially with improved quality of life [35]. Given the risk of medication errors with multiple medications, a wider consideration is that the availability of formal and informal caregivers (who may help with medicines management tasks) is projected to decline [36]. For deprescribing to become mainstream in the very old, we need to know more about its long-term outcomes (and patient-orientated outcomes at that), how best to go about it and what the patients’ viewpoints are. With the COVID-19 pandemic now forcing the development of emergency care plans for older patients, such deprescribing conversations (including within the context of advanced care plans) have never been more important.

### Further research

If preventative medicines of uncertain benefit such as statins were continued in the wider 95-year-old population, future research could investigate: (1) what are patients’ views of stopping preventative medicines at 95—and if there are views on this—do certain patient groups have more concerns about stopping preventative medicines than others, and why? (2) Which medicines do 95-year-olds perceive as most important? (3) In which patient groups are preventative medicines of questionable benefit continued, and in which are they stopped? Understanding these issues may help to focus the deprescribing agenda in the very old and make it more patient-centred.

## Conclusion

In summary, this study examined medication prescription in a cohort of 95-year-olds and found that preventative medications with unclear evidence of benefit were frequently prescribed. Future research in a larger clinical dataset could investigate this preliminary trend, which suggests that evidence to inform preventative medication prescription might be helpful in the very old.

## Electronic supplementary material

Below is the link to the electronic supplementary material.

Supplementary Material 1

Supplementary Material 2
