# Publisher Correction to: Prescribing at 95 years of age: cross-sectional findings from the Newcastle 85+ study

**DOI:** 10.1007/s11096-022-01500-w

**Published:** 2022-10-31

**Authors:** Laurie E. Davies, Andrew Kingston, Adam Todd, Barbara Hanratty

**Affiliations:** 1 Population Health Sciences Institute, Newcastle University, Newcastle upon Tyne, UK; 2 School of Pharmacy, Newcastle University, Newcastle upon Tyne, UK

## Publisher Correction to: International Journal of Clinical Pharmacy (2022) 44:1072–1077 https://doi.org/10.1007/s11096-022-01454-z

The article was published without the keywords “Drug prescription, aged 80 and over, primary care”.

In “Abstract” section, “Conclusions” is corrected as “Conclusion”.

The heading sizes for “Aim” and “Ethics approval” are corrected.

The sentence “In summary, this study examined medication…” till “… prescription might be helpful in the very old.” should be placed under the “Conclusion” section.

The section “Conflict of interest” is corrected as “Conflicts of interest”.

The original article has been corrected.

