# Supplementary material for: Prescribing at 95 years of age: cross-sectional findings from the Newcastle 85+ study
Source: Int J Clin Pharm. 2022 Jul 30;44(4):1072–7. doi: 10.1007/s11096-022-01454-z (PMC9362142; doi:10.1007/s11096-022-01454-z)

**Article title**

# Prescribing at 95 years of age: cross-sectional findings from the Newcastle 85+ Study

**Journal name**

International Journal of Clinical Pharmacy

**Author names and affiliations**

Laurie E Davies[a], Andrew Kingston[a], Adam Todd[b], Barbara Hanratty[a]

a. Population Health Sciences Institute, Newcastle University, Newcastle upon Tyne, United Kingdom

b. School of Pharmacy, Newcastle University, Newcastle upon Tyne, United Kingdom

**Corresponding author e-mail address**

laurie.davies@newcastle.ac.uk

# Online Resource 1: Recruitment and retention in the Newcastle 85+ Study

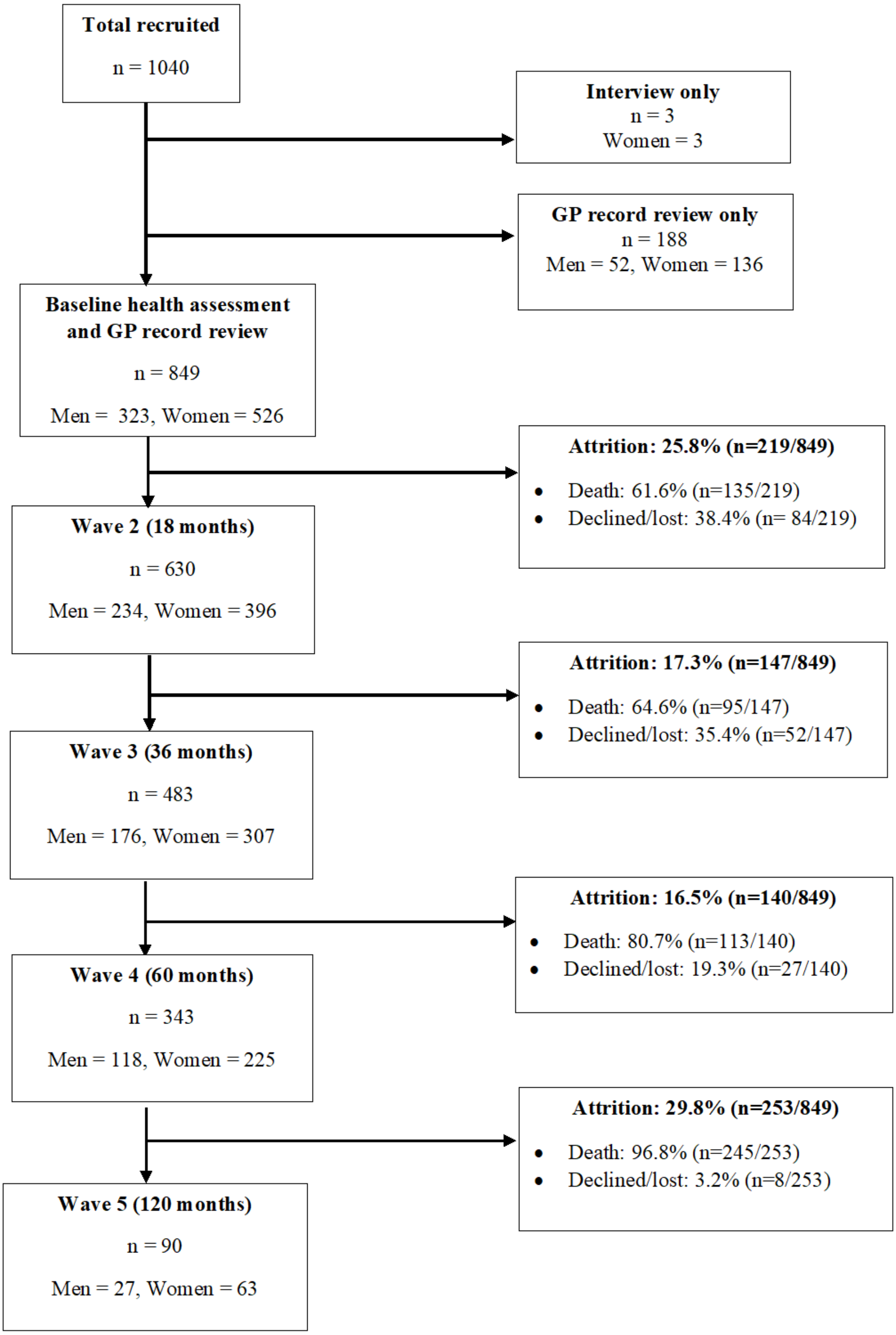

Supplement: Supplementary file 1 — Supplementary Material 1 [file 11096_2022_1454_MOESM1_ESM.pdf]
