# Supplementary material for: Prescribing at 95 years of age: cross-sectional findings from the Newcastle 85+ study
Source: Int J Clin Pharm. 2022 Jul 30;44(4):1072–7. doi: 10.1007/s11096-022-01454-z (PMC9362142; doi:10.1007/s11096-022-01454-z)
Supplement: Supplementary file 2 — Supplementary Material 2 [file 11096_2022_1454_MOESM2_ESM.pdf]

Article title

Prescribing at 95 years of age: cross-sectional findings from the Newcastle 85+ Study

Journal name

International Journal of Clinical Pharmacy

Author names and affiliations

Laurie E Davies[a], Andrew Kingston[a], Adam Todd[b], Barbara Hanratty[a]

[a.] Population Health Sciences Institute, Newcastle University, Newcastle upon Tyne, United Kingdom

[b.] School of Pharmacy, Newcastle University, Newcastle upon Tyne, United Kingdom

Corresponding author e-mail address

laurie.davies@newcastle.ac.uk

Online Resource 2: Prescribed items excluded from analysis

Prescribed item	BNF code
Stoma products	10800
Peak flow meters, inhaler devices and nebulisers	30150
Hypodermic equipment (excluding lancets)	60113
Diagnostic and monitoring agents for diabetes (including lancets)	60160
Ring/shelf pessary	70110
Electrolytes and water - water for injections	90221
Wound dressings	131300
Tubular bandages, compression hosiery and applicators	131310
Diphtheria vaccine	140404
Influenza vaccine	140410
Typhoid vaccine	140419
Pneumococcal vaccine	140420
Urinary catheter/sheath/leg bag	180500
Anal plug for bowel incontinence	180501
Truss - elastic band	180600
Borderline substances - food	180700
Syringe for injection	180800
KY jelly	180801
Sharps bin	180802
Gloves	180803
